# Multispecies emergence of dual *bla*_KPC/NDM_ carbapenemase-producing Enterobacterales recovered from invasive infections in Chile

**DOI:** 10.1128/aac.01205-24

**Published:** 2024-12-05

**Authors:** Ana M. Quesille-Villalobos, Camila Solar, Jose R. W. Martínez, Lina Rivas, Valeria Quiroz, Ana M. González, Roberto Riquelme-Neira, Juan A. Ugalde, Anne Peters, Oscar Ortega-Recalde, Rafael Araos, Patricia García, Francois Lebreton, Jose M. Munita, Lorena Diaz

**Affiliations:** 1 Genomics & Resistant Microbes group (GeRM), Instituto de Ciencias e Innovación en Medicina (ICIM), Facultad de Medicina, Clínica Alemana, Universidad del Desarrollo 441184 , Santiago, Chile; 2 Multidisciplinary Initiative for Collaborative Research on Bacterial Resistance (MICROB-R), Santiago, Chile; 3 Facultad de Medicina Veterinaria y Agronomía, Universidad de Las Américas 28059 , Concepción, Chile; 4 Center for Bioinformatics and Integrative Biology, Facultad de Ciencias de la Vida, Universidad Andres Bello 28087 , Santiago, Chile; 5 Departamento de Morfología, Facultad de Medicina, Universidad Nacional de Colombia 206200 , Bogotá, Colombia; 6 Departamento de Laboratorios Clínicos, Escuela de Medicina, Pontificia Universidad Catolica de Chile 28033 , Santiago, Chile; 7 Multidrug-Resistant Organism Repository and Surveillance Network, Walter Reed Army Institute of Research 8394 , Silver Spring, Maryland, USA; Johns Hopkins University School of Medicine, Baltimore, Maryland, USA

**Keywords:** dual carbapenemase-producing CRE, carbapenem-resistant Enterobacterales, Enterobacterales, antibiotic resistance

## Abstract

Carbapenemase-producing carbapenem-resistant Enterobacterales (CP-CRE) represent a significant global threat. The emergence of dual CP-CRE is particularly alarming, as they can potentially compromise the efficacy of newer antibiotics, further decreasing therapeutic alternatives. Herein, we report the emergence of multiple species of CP-CRE recovered from invasive infections in Chile that simultaneously harbor *bla*_KPC_ and *bla*_NDM_ and provide an in-depth genomic characterization of these worrisome pathogens. We collected carbapenem-resistant Enterobacterales (CRE) isolates from invasive infections over a 4-year period, across 11 healthcare centers in Chile. Bacterial species and the presence of carbapenemase genes were confirmed using MALDI-TOF and PCR assays, respectively. Antimicrobial susceptibility testing was conducted through disk diffusion and broth microdilution methods. Dual CP-CRE isolates were subjected to short- and long-read whole genome sequencing to perform a detailed genomic characterization of the isolates and of the mobile genetic elements harboring the enzymes. From a total of 1,335 CRE isolates, we observed an increase in the prevalence of CP-CRE, from 11% in 2019 to 38% in 2022. A total of 11 dual CP-CRE isolates were recovered, all of them harboring *bla*_KPC_ and *bla*_NDM_. Species corresponded to *Escherichia coli* (*n* = 6), *Klebsiella pneumoniae* (*n* = 2), *Klebsiella oxytoca* (*n* = 2), and *Citrobacter freundii* (*n* = 1). Dual CP-CRE isolates exhibited resistance to all tested β-lactams except for cefiderocol. The *bla*_KPC_ and *bla*_NDM_ encoding genes were located on independent plasmids. Platforms harboring *bla*_KPC_ were diverse and included IncN, IncF, and IncFIB plasmids. In contrast, *bla*_NDM-7_ was only found on fairly conserved IncX3 plasmids. We report that a rapid increase of CP-CRE in Chile, alongside with the emergence of multiple bacterial species of CP-CRE co-harboring *bla*_KPC-2/3_ and *bla*_NDM-7_, underscores a critical public health challenge. Our data suggest that the dissemination of *bla*_NDM-7_ was predominantly facilitated by IncX3 plasmids, whereas the spread of *bla*_KPC_ involved multiple plasmid backbones. Active surveillance and genomic monitoring are critical to inform public policy and curtail the spread of these highly resistant pathogens.

## INTRODUCTION

Carbapenem-resistant Enterobacterales (CRE) are a major concern worldwide (1). The most common mechanism of carbapenem resistance is the production of enzymes that efficiently hydrolyze carbapenems, known as carbapenemases. These enzymes are usually classified as class A, serine-β-lactamases (e.g., KPC); class B, metallo-β-lactamases (MBLs) (e.g., NDM); and class D carbapenemases (e.g., OXA-48 like) (2). Since their first description, carbapenemase-producing carbapenem-resistant Enterobacterales (CP-CRE) have been found globally, with carbapenemase-encoding genes disseminating across different bacterial species and environments using multiple mobile genetic elements (3).

While most CP-CRE harbor a single carbapenemase gene, isolates carrying multiple carbapenemase genes (dual CP-CRE) have been well reported (4–10). This genotype is particularly concerning as it could potentially impair the activity of newer molecules such as cefiderocol (FDC), further decreasing our already limited therapeutic options. Worrisomely, a surge of dual CP-CRE has been described in some Latin American countries, with the majority of isolates co-harboring *bla*_KPC/NDM_ (4, 8, 10–12). Genomic data regarding the mobile genetic elements underlying this epidemiological change in the region are limited. In this study, we report the multispecies emergence of dual CP-CRE co-harboring *bla*_KPC_ and *bla*_NDM_ (*bla*_KPC/NDM_) recovered as part of an ongoing prospective collection of invasive CRE from 11 healthcare centers in Chile and provide a detailed genomic characterization of these highly resistant pathogens.

## MATERIALS AND METHODS

### Collection of isolates

A total of 1,335 Enterobacterales resistant to at least one carbapenem (ertapenem [ETP], meropenem [MEM], and imipenem [IPM]) were recovered from blood (62%), bone tissue (13%), sterile fluids (10%), biopsies (5%), and other sources (10%) of hospitalized patients in 11 Chilean healthcare centers between January 2019 and December 2022. All isolates were sent to a central laboratory for further characterization. Bacterial species were confirmed by MALDI-TOF (Microflex LT, Bruker Daltonik), and the presence of carbapenemase-encoding genes (i.e., *bla*_KPC_, *bla*_VIM_, and *bla*_NDM_) was assessed by multiplex PCR (13). Carbapenemase enzyme production was further confirmed by an immunochromatographic assay (NG-Test CARBA 5).

### Antimicrobial susceptibility testing

Antimicrobial susceptibility to ceftriaxone (CRO), cefotaxime (CTX), ceftazidime (CAZ), cefepime (FEP), piperacillin-tazobactam (TZP), ampicillin-sulbactam (SAM), ETP, IPM, MEM, amikacin (AN), gentamicin (GM), ciprofloxacin (CIP), and trimethoprim-sulfamethoxazole (SXT) was evaluated using the disk diffusion method (Oxoid) following Clinical & Laboratory Standards Institute (CLSI) guidance (14). Additionally, the minimum inhibitory concentration (MIC) to all three carbapenems and FDC was determined in duplicate for dual CP-CRE using broth microdilution (BMD) as per CLSI recommendations (14). Quality control strains *E. coli* ATCC 25922 and *P. aeruginosa* ATCC 27853 were included in all assays.

### Whole genome sequencing and genomic characterization

Genomic DNA of all 11 dual CP-CRE isolates was purified from fresh cultures with the DNeasy Blood & Tissue kit (Qiagen). DNA concentration was determined with the Qubit dsDNA HS Assay in a Qubit 2.0 fluorometer (Thermo Fisher Scientific). Genomic libraries were prepared using Illumina DNA prep kit (Illumina) with IDT 10 bp UDI indexes and sequenced on Illumina NextSeq 2000, producing 2 × 150 bp reads. All dual CP-CRE isolates were also long-read sequenced using the Oxford Nanopore Technologies (ONT) Ligation Sequencing Kit (SQK-NBD114.24) with NEBNext Companion Module (E7180L) as per the manufacturer’s specifications. Samples were run on a MinION Mk1B (Oxford Nanopore) using R10.4.1 flow cells. Read quality was evaluated using FASTQC (v.0.11.9) (15) and MultiQC (v.10.1) (16). Read pairs with a quality score above 30 were trimmed with Trimmomatic (v0.39) (17). Hybrid *de novo* genome assemblies using both short- and long-reads were performed using SPAdes v3.13.0 (18); the quality of assemblies was assessed with QUAST v5.0 (19). *In silico* determination of sequence types (ST) was performed using MLST (v2.19.0) (20). Antimicrobial resistance genes were identified using AMRFinder (v3.11.26) (21) and annotated with CARD (v.RGI 6.0.2) (22). All genomes were annotated with Bakta (v1.0) (23) and visualized using Proksee (v1.0) to identify contigs harboring carbapenemase encoding genes and circularize plasmids (24). Geneious Prime (v2023.2.1) and Easyfig (v2.2.5) were used to analyze the homology and synteny of carbapenemase-harboring plasmids (25). Plasmid typing and the identification of incompatibility group were performed with MOB-suite v3.1.4 mob_typer module with default parameters (26). Single nucleotide polymorphisms (SNPs) were assessed between isolates from the same species recovered from a single patient using Snippy v4.6.0 (27).

## RESULTS

### The frequency of CP-CRE and dual CP-CRE has increased over time in Chile

**Fig 1:**
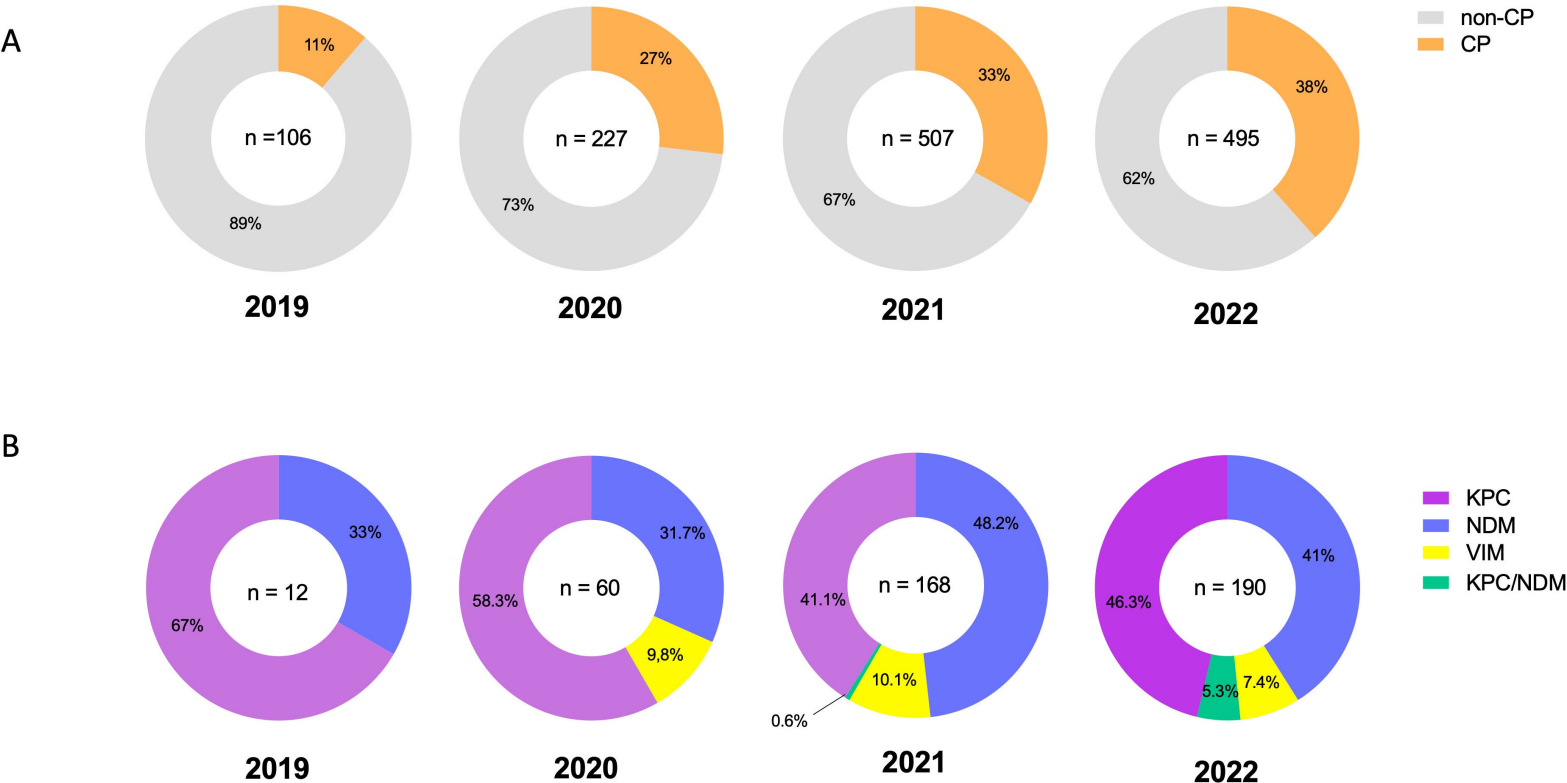
Increase of carbapenemases in CRE in Chile over time. (A) Detection of carbapenemases in CRE between 2019 and 2022; non-carbapenemase producers (non-CP) and carbapenemase producers (CP) are indicated by year of isolation. (B) Type of carbapenemases detected in CP-CRE isolates by year of isolation.

A total of 1,335 invasive CRE isolates were collected from 2019 to 2022 and recovered from deep-seated infections such as blood (62%), bone tissue (13%), sterile fluids (10%), biopsies (5%), and others (10%). The most frequently identified species was *Klebsiella pneumoniae* (*n* = 1,002), followed by *Enterobacter cloacae* complex (*n* = 167), *Escherichia coli* (*n* = 45), *K. oxytoca* (*n* = 21), and *Citrobacter* spp. (*n* = 14). Overall, 67% of isolates recovered during the study period corresponded to non-carbapenemase-producing CRE. However, the landscape of CP-CRE changed over time in the country, increasing from only 11% of isolates in 2019 to 38% in 2022 (Fig. 1A). The most commonly detected carbapenemase genes were *bla*_KPC_ (46%) and *bla*_NDM_ (42%).

The first detection of a dual CP-CRE harboring *bla*_KPC/NDM_ in our collection occurred in November 2021 and corresponded to a *K. oxytoca*. Then, ten additional isolates were recovered during 2022, representing 5% of the total amount of CP-CRE isolated that year (Fig. 1B). Isolates co-harboring *bla*_KPC/NDM_ were obtained across four healthcare centers and encompassed four different bacterial species, including *E. coli* (*n* = 6), *K. pneumoniae* (*n* = 2), *K. oxytoca* (*n* = 2)*,* and *C. freundii* (*n* = 1) (Table 1).

**TABLE 1:** Characteristics of DCP-CRE isolates^*a*^

Patient	ID	Species	Hospital	Date (mo-yr)	Source	ST	ꞵ-lactamase genes	Other resistance genes	Plasmid size (bp)/replicon family		MIC (µg/mL)				Genome accession number
									*bla*_KPC_	*bla*_NDM_	MEM	IPM	ATM	FDC	
1	SCL12139	*K. oxytoca*	A	Nov-2021	Blood	242	*bla*_KPC-2_, *bla*_NDM-7_ *bla*_OXY-5-1_	*aac(6')-Ib, aadA16, sul1, dfrA27, fosA, oqxA, oqxB9, qnrB6*	52,137/IncN	45,711/IncX3	>32	>32	>32	2	JBFDWC000000000
2	SCL13636	*K. pneumoniae*	B	Apr-2022	Bone	25	*bla*_KPC-3_, *bla*_NDM-7_ *bla*_CTX-M-15_, *bla*_SHV-110_, *bla*_TEM-1_	*aac (3)-Iie, aac(6')-Ib, aadA2, aph(3'')-Ib, aph (6)-Id, armA, sul1, sul2, dfrA12, dfrA14, fosA6, mph(E), msrE, oqxA7, oqxB17, qnrB1, qnrB19*	179,879/IncFIB; IncFII; CoIRNAI_rep_cluster_1857	57,977/IncX3	>32	>32	>32	4	JBEHHG000000000
3	SCL13674	*E. coli*	B	Apr-2022	Blood	295	*bla*_KPC-2_, *bla*_NDM-7_	*aac(6')-Ib, aadA16, sul1, dfrA27, qnrB6*	52,667/IncN	46,160/IncX3	>32	>32	>32	0.5	JBEHHF000000000
	SCL13680	*E. coli*	B	Apr-2022	Blood	295	*bla*_KPC-2_, *bla*_NDM-7_	*aac(6')-Ib, aadA16, sul1, dfrA27, qnrB6*	58,960/IncN	46,160/IncX3	>32	>32	>32	1	JBEHHE000000000
4	SCL13933	*E. coli*	C	Apr-2022	Blood	361	*bla*_KPC-2_, *bla*_NDM-7_	*aac(6')-Ib, aadA16, sul1, dfrA27, qnrB6*	58,134/IncN	46,161/IncX3	>32	>32	>32	1	JBEHHD000000000
	SCL13934	*E. coli*	C	Apr-2022	Blood	361	*bla*_KPC-2_, *bla*_NDM-7_	*aac(6')-Ib, aadA16, sul1, dfrA27, qnrB6*	57,092/IncN	46,161/IncX3	>32	>32	>32	0.5	JBEHHC000000000
	SCL13936	*E. coli*	C	Apr-2022	Blood	361	*bla*_KPC-2_, *bla*_NDM-7_	*aac(6')-Ib, aadA16, sul1, dfrA27, qnrB6,*	57,093/IncN	46,159/IncX3	>32	>32	>32	1	JBEHHB000000000
	SCL13938	*E. coli*	C	Apr-2022	Blood	361	*bla*_KPC-2_, *bla*_NDM-7_	*aac(6')-Ib, aadA16, sul1, dfrA27, qnrB6,*	57,093/IncN	46,160/IncX3	>32	>32	>32	1	JBEHHA000000000
5	SCL14225	*C. freundii*	A	June-2022	Bone	22	*bla*_KPC-2_, *bla*_NDM-7_ *bla*_CMY-48_, *bla*_CTX-M-3_	*aac(6')-Ib, aadA1, aadA2, armA, catB3, sul1, dfrA1, dfrA12, mph(E), msrE*	104,660/ND	48,839/IncX3	>32	>32	>32	8	JBEHGZ000000000
6	SCL16432	*K. oxytoca*	A	Dec-2022	Blood	242	*bla*_KPC-2_, *bla*_NDM-7_ *bla*_OXY-5-1_*, bla*_TEM_	*aac(6')-Ib-cr5, aadA16, sul1, dfrA27, fosA, oqxB,*	60,431/IncN	45,713/IncX3	>32	>32	>32	2	JBFDWB000000000
7	SCL16743	*K. pneumoniae*	D	Dec-2022	Blood	45	*bla*_KPC-2_, *bla*_NDM-7_ *bla*_CTX-M-15_, *bla*_TEM-1_	*fosA, oqxB19, oqxA11*	98,512/IncFIB; IncFII	25,272/IncX3	>32	>32	>32	2	JBEHGY000000000

^*a*^ MIC, minimal inhibitory concentration; MEM, meropenem; IPM, imipenem; ATM, aztreonam; FDC, cefiderocol; ND, not determined.

### Phenotypic and genomic characterization of dual CP-CRE isolates

The 11 dual CP-CRE isolates exhibited resistance to all tested β-lactams except for FDC (see below). A majority of them were also resistant to CIP and SXT, and susceptibility to aminoglycosides was variable (Table S1). BMD revealed all dual CP-CRE isolates exhibited MICs to ETP, IPM, and MEM ≥64 µg/mL. In contrast, 10 of 11 isolates remained susceptible to FDC, with MICs ranging from 0.5 to 4 µg/mL. The remaining strain corresponded to a *C. freundii* with a MIC of 8 µg/mL, categorized as intermediate as per CLSI (2023) breakpoints (Table 1), and whose whole genome analysis did not reveal any genetic change previously associated with FDC resistance.

Table 1 summarizes the *in silico* MLST analyses per species. Both *K. oxytoca* strains were recovered in the same hospital from two patients more than 12 months apart, and both belonged to an ST242 lineage. In contrast, the two *K. pneumoniae* were recovered from different healthcare institutions and belonged to distinct lineages: ST25 and ST45. The only *C. freundii* belonged to an ST22 lineage (Table 1). Out of the six dual CP *E. coli*, four ST361 isolates were recovered from the bloodstream of a single patient. The remaining two belonged to ST295 and were obtained from the bloodstream of a patient in a different hospital. To evaluate the genomic relatedness between *E. coli* isolates obtained from the same individual, an SNP analysis was performed using each patient’s first isolate as the reference. We found 63 SNPs of difference between isolates from patient 3 and 94-97 SNPs between *E. coli* isolates recovered from patient 4, revealing a significant variation among strains (28) (Table S3; Fig. S1).

Genomic analyses confirmed the presence of *bla*_KPC_ and *bla*_NDM_ in the 11 dual CP-CRE isolates, all harboring a *bla*_NDM- 7_ allele. In the case of *bla*_KPC_, ten isolates carried *bla*_KPC-2_, and one *K. pneumoniae* contained a *bla*_KPC-3_ allele (Table 1). Our resistome analysis demonstrated all strains of *K. pneumoniae*, *K. oxytoca*, and *C. freundii* harbored additional β-lactamases, including *bla*_OXY-5-1_, *bla*_CTX-M-15_, and *bla*_CTX-M-3_ (Table 1).

### Mobile genetic elements containing *bla*_KPC_ and *bla*_NDM_ in dual CP-CRE isolates

**Fig 2:**
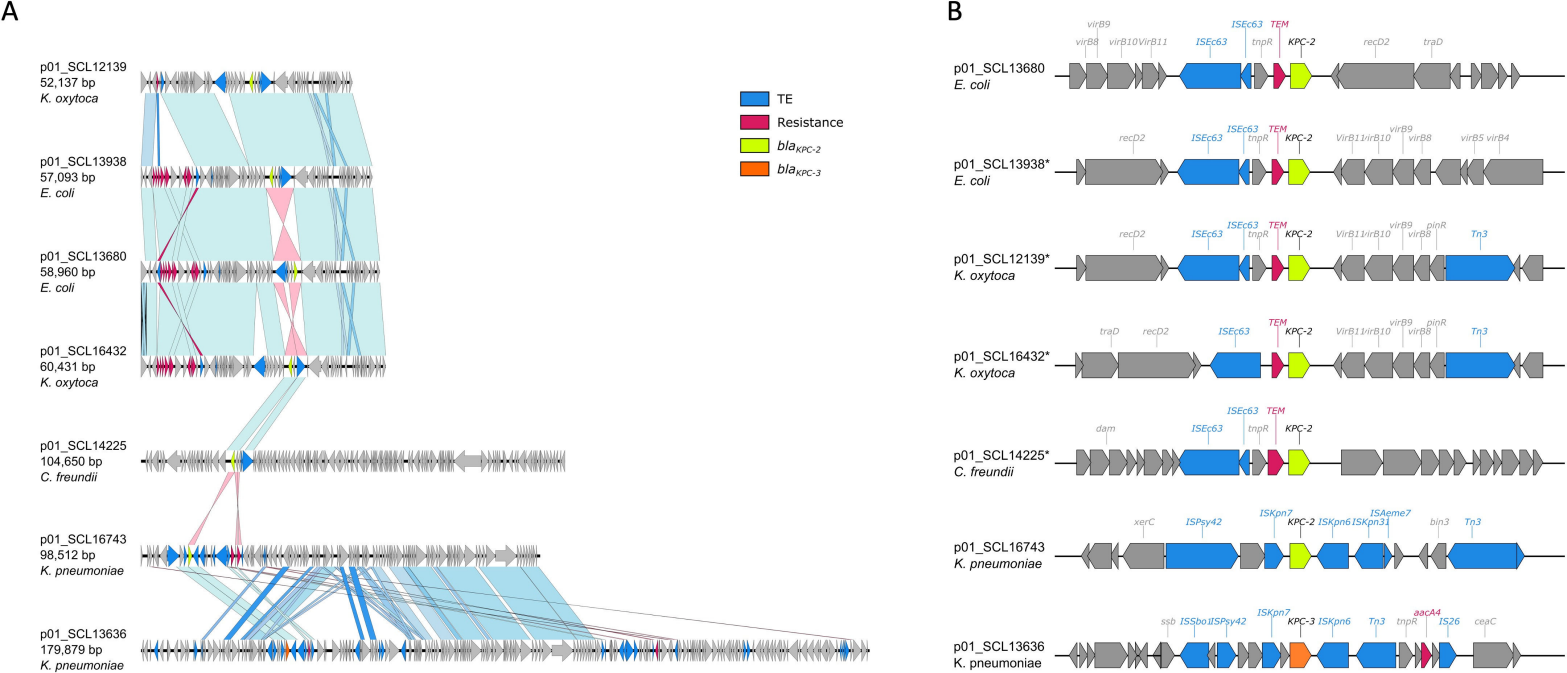
Plasmids carrying *bla*_KPC_ in dual CP-CRE isolates. (A) Alignment of plasmids carrying *bla*_KPC-2_ gene in dual CP-CRE isolates. (B) Genetic context of *bla*_KPC-2_, and *bla*_KPC-3_, flanking regions in dual CP-CRE isolates. Genes without information correspond to hypothetical proteins.

**Fig 3:**
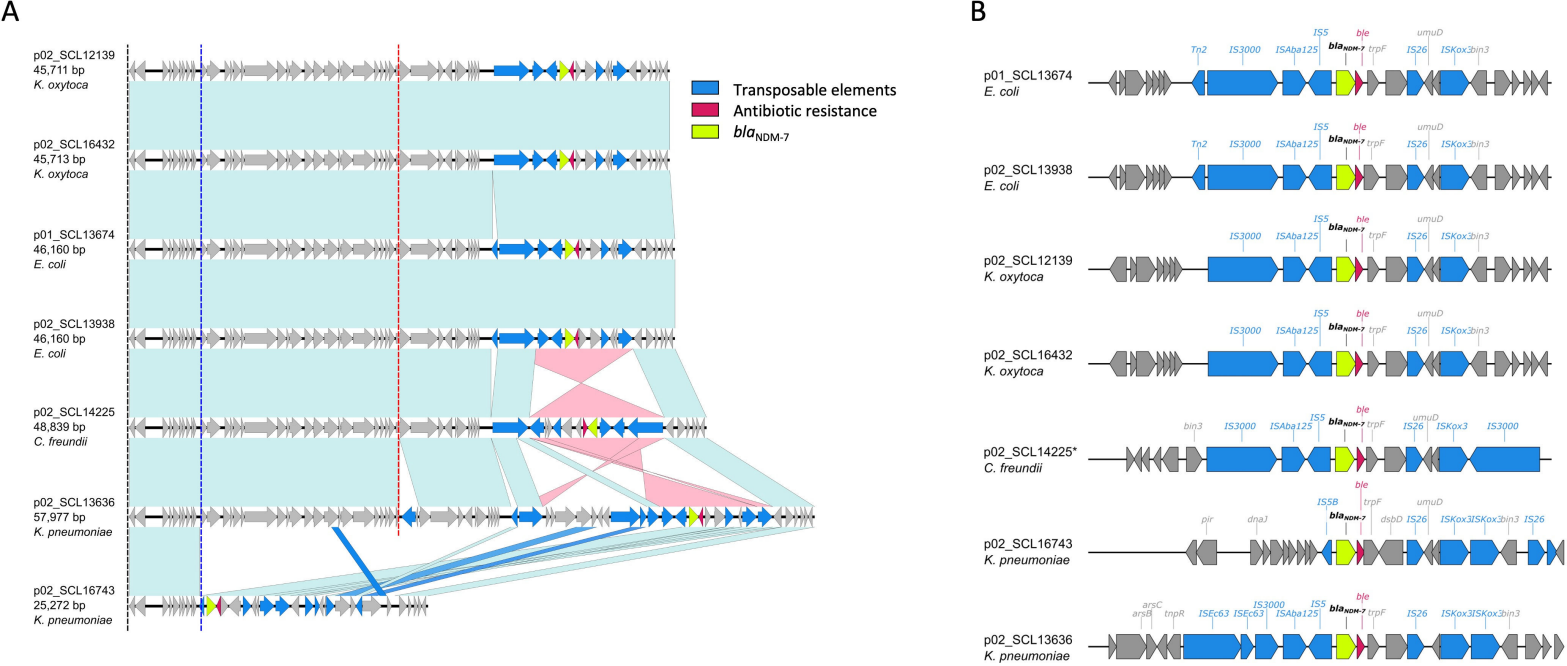
Plasmids carrying *bla*_NDM_ in dual CP-CRE isolates. (A) Alignment of plasmids carrying *bla*_NDM-7_ gene in dual CP-CRE isolates. (B) Genetic context of *bla*_NDM-7_, flanking regions in dual CP-CRE isolates. Genes without information correspond to hypothetical proteins.

The closed genomes of the 11 isolates demonstrated that both *bla*_KPC_ and *bla*_NDM_ genes were carried on independent plasmids (Fig. 2 and 3).

The size of plasmids harboring *bla*_KPC-2_ ranged from 52 to 180 Kbp and belonged to three incompatibility groups: IncN, IncF, and IncFIB. The four IncN plasmids were observed in *E. coli* and *K. oxytoca* and exhibited a sequence identity >93% over their entire length (Table 1; Fig. 2A). The incompatibility group of a 104 Kbp plasmid carrying *bla*_KPC-2_ in the *C. freundii* isolate was not able to be determined, and a multiple sequence alignment with the rest of the plasmid studied showed that it only shared a 6.9% sequence similarity (Fig. 2A). Finally, two *bla*_KPC_-containing plasmids were found in *K. pneumoniae* isolates: one harbored *bla*_KPC-2_ (IncF, IndFIB; 98 Kbp) and the other a *bla*_KPC-3_ allele (IncF, IncFIB, and ColRNAI_rep_cluster_1857; 179 Kbp). Plasmids obtained from *K. pneumoniae* only shared 30% of nucleotide sequence identity (Fig. 2A). Despite the differences observed among the backbones of *bla*_KPC_-containing plasmids, the direct genetic environment of *bla*_KPC-2_ was conserved. Specifically, *bla*_KPC-2_ was located downstream of a truncated *bla*_TEM-1_ gene and an insertion sequence homologous to ISEc6, and it was harbored by a non-Tn*4401* element NTE_KPC_ inserted in the *Tra* operon required for conjugation (Fig. 2B).

A comparison with publicly available databases using our *bla*_KPC-2_-containing *E. coli* plasmid p01_SCL13938 revealed a high sequence similarity (99%) with *bla*_KPC-2_ plasmids previously obtained from *K. pneumoniae* in Chile (11), Portugal, and South Korea (Table S2). Interestingly, the only *bla*_KPC-3_-harboring plasmid in our collection (p01_13636) exhibited a high sequence identity (99% over 85–87% of its total length) with a plasmid previously recovered from a non-carbapenemase-producing *K. pneumoniae* isolated in Chile from a cancer patient with persistent bacteremia that failed imipenem therapy and developed resistance to carbapenems *in vivo* (accession numbers CP093482.1 and CP061833.1, Table S2) (29).

By contrast, all plasmids harboring *bla*_NDM-7_ belonged to the same IncX3 incompatibility group and exhibited sizes ranging from 25 to 58 Kbp. As shown in Fig. 3A, a multiple nucleotide comparison revealed a high degree of similarity among *bla*_NDM_-harboring plasmids across different bacterial species from our collection. Indeed, plasmids from *K. oxytoca* (p02_SCL12139 and p02_SCL16432), *E. coli* (p01_SCL13674 and p02_SCL13938), and *C. freundii* (p02_SCL14425) were almost identical, exhibiting >99% similarity. On the other hand, plasmids from *K. pneumoniae* isolates (p02_SCL13636 and p02_SCL16743) showed important differences in their size and gene content (Fig. 3A). As previously described in Canada and Germany (30, 31), the *bla*_NDM-7_-containing region was inserted downstream of the serine resolvase. Additionally, *bla*_NDM-7_ was carried by an IS*3000*-ΔIS*Aba125*-IS*5*-ΔIS*Aba125-bla*_NDM-7_-*ble*_MBL_-*trpF-dsbC*-IS*26*--Δ*umuD*-IS*Kox3* genetic element (Fig. 3B), a similar structure as that of the *bla*_NDM-4-_, *bla*_NDM-5-_, and *bla*_NDM-7_-containing elements reported previously (30–33). We observed insertions of IS*Ec63* (1 *K*. *pneumoniae*) or IS*2* upstream of this element (2 *E. coli* isolates) and deletions of IS*3100* and IS*Aba125* (1 *K*. *pneumoniae*), suggesting that additional genetic rearrangements may occur in these plasmids. A query against the NCBI database using the *bla*_NDM-7_ plasmid from *E. coli* (p02_SCL13938) showed >99% sequence similarity with plasmids previously observed in *E. coli* harboring *bla*_NDM-5_ isolated in China (Table S2). A high sequence identity (100%) along the full-length sequence of p02_SCL16432 was also observed with plasmids carrying *bla*_NDM-7_ obtained from *E. coli* previously reported in the Arabian Peninsula (34).

## DISCUSSION

Our data from a multicenter prospective collection of CRE isolates causing invasive infections in Chile suggest that the landscape of CRE in the country is rapidly changing, with a drastic surge of CP-CRE over the years, increasing ~250% from 2019 to 2022 (Fig. 1). In addition, we describe the multispecies emergence of Enterobacterales co-harboring *bla*_KPC2/3_ and *bla*_NDM-7_ in different healthcare centers across the country. Furthermore, we provide detailed genomic analyses suggesting that while *bla*_NDM-7_ is being spread in IncX3 plasmids, *bla*_KPC_ genes are being spread via a NTE_KPC_ inserted into a variety of plasmid backbones.

The emergence of dual CP-CRE is becoming an increasing concern, with reports from different regions, including Asia, Europe, Africa, North America, and South America (5, 35–37). Most dual CP-CRE reports correspond to *K. pneumoniae* (~70%), followed by *E. coli*, which accounts for 10–15% (35). Multiple enzyme combinations have been described, with frequencies varying by geographical distribution and bacterial species (35). Interestingly, a recent report summarizing the literature on multiple carbapenemase production suggested the large majority of publications come from Asia, with less than 5% of manuscripts representing the Americas (35). Regarding enzyme combinations, co-production of *bla*_OXA_ plus *bla*_NDM_ accounts for almost two of every three reports (35), with multiple other combinations described (36, 38, 39). Albeit less frequently, the combination of *bla*_KPC_ plus *bla*_NDM_, as observed in our isolates, has also been reported in several regions, including South America (40, 41). Indeed, a recent manuscript from Argentina describing 82 dual CP-CRE co-producing *bla*_KPC_ plus a metalloenzyme reported that 93% of cases exhibited a combination of *bla*_KPC_ plus *bla*_NDM_ (42), most of which (77 out of 82) corresponded to *K. pneumoniae*. In contrast to our data, most *bla*_NDM_ alleles found in combination with other carbapenemases corresponded to *bla*_NDM-1_ or *bla*_NDM-5_, with very sporadic reports of *bla*_NDM-7_, as found in our multispecies collection (32, 43–47).

Regarding genomic lineages, our dual CP-CR *E. coli* belonged to ST361 and ST295. Interestingly, ST361 was reported by the (48) surveillance as a frequent carrier of *bla*_NDM-5_ and a common cause of invasive infections in Europe (48). Moreover, the emergence of ST361 *E. coli* co-harboring *bla*_NDM-5_, *bla*_KPC-3_, and *bla*_CTX-M-15_ was recently reported in patients evacuated from Ukraine. Notably, such strains also exhibited resistance to FDC and ATM/AVI due to a PBP3 YRIN insertion (49). Our dual CP-CR *K. pneumoniae* belonged to ST25 and ST45, none of which have been widely reported as dual CP. Interestingly, Veloso et al. recently analyzed the genomes of 10 CR-*K. pneumoniae* from Chile, reporting isolates from ST25 and ST45 that appear to have recently acquired *bla*_NDM-7_ (11). Moreover, previous data from invasive CR-*K. pneumoniae* suggested ST25 accounted for a large proportion of isolates circulating in Chile, but carbapenemase production was extremely infrequent before 2021 (50). On the other hand, CR-*K. pneumoniae* isolates from the ST45 lineage have been reported as part of a *bla*_NDM-1_ neonatal outbreak in China (51) and were found as an emerging *bla*_NDM-7_-producing lineage in Chile in 2021 (50).

Our results demonstrated the *bla*_NDM-7_ gene was consistently harbored on IncX3 plasmids previously described as conjugative between different species (36, 52–56) (Fig. 2A). While IncX3 plasmids are well-known to harbor NDM enzymes, the *bla*_NDM-7_ allele continues to account for a minority of them. In particular, data from South America suggest most CP-CRE isolates harboring metalloenzymes contain either *bla*_NDM-1_ or *bla*_NDM-5_ (57). In the case of *bla*_KPC_, our plasmids were more diverse and grouped into different incompatibility groups. Interestingly, the *bla*_KPC-2_ p01_SCL14225 plasmid exhibited 85% query coverage and 99% identity to pRHBSTW-00697, a plasmid obtained from a *C. freundii* strain isolated from livestock microbiota in the United Kingdom that did not harbor any antimicrobial resistance traits (GenBank accession no. CP056338) (58). In contrast to pRHBSTW-00697, our *bla*_KPC-2_-harboring plasmid had a ~6,700 bp insertion that contained *bla*_KPC-2_, *bla*_TEM_, and an ISEc63 Tn3 family transposon. While the typical genetic element that harbors *bla*_KPC_ is a Tn3-based transposon (59), our *bla*_KPC_ plasmids were similar to those recently described in a multispecies outbreak of CP-CRE that contained a nonclassical Tn*4401* transposon element NTE_KPC_ (60), which are primarily reported in lineages different from ST258 *K. pneumoniae* or other Enterobacterales (59). Interestingly, the only *bla*_KPC-3_-harboring plasmid observed in our dual-CP-CRE collection was identified in an ST25 *K. pneumoniae* and shared high coverage (87%) and 99.9% identity with a plasmid previously reported in a non-CP-CR *K. pneumoniae* recovered from the bloodstream of a neutropenic patient in Chile (29).

The emergence of dual CP-CRE is extremely concerning since it may limit our already scarce therapeutic options, particularly against metalloenzymes (7). Our *in vitro* susceptibility testing revealed all dual CP-CRE isolates from our collection exhibited high-level resistance to carbapenems and aztreonam (Table 1), which ought to be expected. Among the newer alternatives, FDC has been shown to retain activity against NDM-producing CRE (61, 62). Our data demonstrated all but one isolate (a *C. freundii* isolate with a MIC of 8 µg/mL) exhibited FDC MICs classified as susceptible, ranging between 0.5 and 4 µg/mL. Interestingly, isolates from our collection harboring additional class A ESBLs (e.g., *bla*_CTX-M-15_, *bla*_CTX-M-3_) tended to have higher FDC MICs (2–4 µg/mL), suggesting non-carbapenemase class A enzymes could play a role in the activity of FDC against CP-CRE.

In summary, the molecular epidemiology of CRE in Chile is rapidly changing from mostly non-carbapenemase producing organisms to the emergence of CP-CRE. Additionally, our data suggest that while the rapid spread of NDM-7 in Chile is being driven by horizontal transmission of IncX3 plasmids, the dissemination of KPC might result from multiple introduction events on plasmids already adapted to Enterobacterales circulating in the country. Continued surveillance of these highly resistant pathogens will be essential to inform strategies to prevent their widespread dissemination and to guide therapeutic decisions.

## Data Availability

All genomes included in this study are publicly available at the U.S. National Center for Biotechnology Information at the Genome Sequence Archive under the BioProject ID PRJNA1044051.

## SUPPLEMENTAL MATERIAL

Supplemental material aac.01205-24-s0001.docx Tables S1 to S3; Figures S1 and S2.

ASM does not own the copyrights to Supplemental Material that may be linked to, or accessed through, an article. The authors have granted ASM a non-exclusive, world-wide license to publish the Supplemental Material files. Please contact the corresponding author directly for reuse.
