# Supplementary material for: Multispecies emergence of dual blaKPC/NDM carbapenemase-producing Enterobacterales recovered from invasive infections in Chile
Source: Antimicrob Agents Chemother. 2024 Dec 5;69(1):e01205-24. doi: 10.1128/aac.01205-24 (PMC11784225; doi:10.1128/aac.01205-24)
Supplement: Supplemental material — Tables S1 to S3; Figures S1 and S2. [file aac.01205-24-s0001.docx]

**SUPPLEMENTARY MATERIAL**

**Multispecies emergence of dual *bla*_KPC/NDM_ carbapenemase-producing Enterobacterales recovered from invasive infections in Chile**

Quesille-Villalobos AM^#a,b^, Solar C^#a,b^, Martínez JRW^a,b^, Rivas L^a,b^, Quiroz V^a,b^, González AM^a,b^, Riquelme-Neira R^b,c^, Ugalde JA^b,d^, Peters A^a,b^, Ortega-Recalde O ^a,f^, Araos R^a,b^, García P^b,e^, Lebreton F^g^ , Munita JM^a,b*^, Díaz L^a,b,f^*.

^a^Genomics & Resistant Microbes group (GeRM), Instituto de Ciencias e Innovación en Medicina (ICIM), Facultad de Medicina Clinica Alemana, Universidad del Desarrollo, Chile.

^b^Multidisciplinary Initiative for Collaborative Research on Bacterial Resistance (MICROB-R), Santiago, Chile.

^c^Facultad de Medicina Veterinaria y Agronomía, Universidad de Las Américas, Concepción, Chile

^d^Center for Bioinformatics and Integrative Biology, Facultad de Ciencias de la Vida, Universidad Andrés Bello, Santiago, Chile.

^e^Departamento de Laboratorios Clínicos, Escuela de Medicina, Pontificia Universidad Católica de Chile, Santiago, Chile.

^f^Departamento de Morfología, Facultad de Medicina, Universidad Nacional de Colombia, Bogotá, Colombia

^g^Multidrug-Resistant Organism Repository and Surveillance Network, Walter Reed Army Institute of Research, Silver Spring, MD

Running title: Emergence of Dual-Carbapenemase Producing Enterobacterales

#: equal contribution

* Correspondence to: Lorena Díaz, lorenadiazortiz@udd.cl

* Alternate correspondence to: Jose M. Munita, josemunita@udd.cl

**Supplementary Table 1.** Antibiotic resistance profile of DCP-CRE isolates.


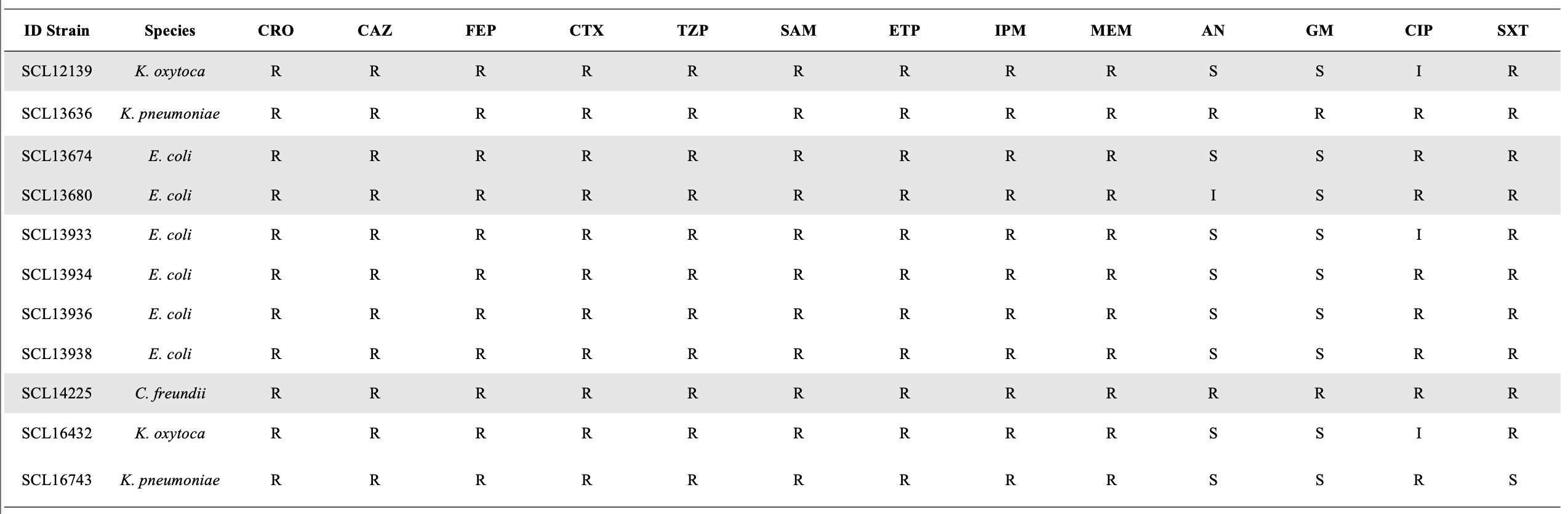


Susceptibilities were determined by disc diffusion and interpreted according to CLSI 2024. CRO= Ceftriaxone; CAZ= Ceftazidime; FEP= Cefepime; CTX= Cefotaxime, TZP= Piperacillin-tazobactam, SAM= Ampicillin-Sulbactam; ETP= Ertapenem; IMP= Imipenem, MEM= Meropenem; AN= Amikacin; GM= Gentamicin; CIP= Ciprofloxacin; SXT= Trimethoprim-Sulfamethoxazole.

**Supplementary Table 2**. BLASTn analysis of Enterobacterales plasmids sequences of 1^st^ isolated from patient.

| **Carbapenemase** | **ID_Plasmid** | **Accession** | [**Max Score**](https://blast.ncbi.nlm.nih.gov/Blast.cgi?CMD=Get&ADV_VIEW=yes&ADV_VIEW=on&ALIGNDB_BATCH_ID=31534727&ALIGNDB_CGI_HOST=blast.st-va.ncbi.nlm.nih.gov&ALIGNDB_CGI_PATH=/ALIGNDB/alndb_asn.cgi&ALIGNDB_MASTER_ALIAS=SD_ALIGNDB_MASTER&ALIGNDB_MAX_ROWS=100&ALIGNDB_ORDER_CLAUSE=seq_evalue%20asc,aln_id%20asc&ALIGNDB_WHERE_CLAUSE=seq_evalue%20is%20not%20null&ALIGNMENTS=100&ALIGNMENT_VIEW=Pairwise&CONFIG_DESCR=ClustMemNbr,ClustComn,Ds,Sc,Ms,Ts,Cov,Eval,Idnt,AccLen,Acc&DATABASE_SORT=0&DESCRIPTIONS=100&DYNAMIC_FORMAT=on&FIRST_QUERY_NUM=0&FORMAT_NUM_ORG=1&FORMAT_OBJECT=Alignment&FORMAT_PAGE_TARGET=&FORMAT_TYPE=HTML&GET_SEQUENCE=yes&I_THRESH=&LINE_LENGTH=60&MASK_CHAR=2&MASK_COLOR=1&NUM_OVERVIEW=100&PAGE=MegaBlast&QUERY_INDEX=0&QUERY_NUMBER=0&RESULTS_PAGE_TARGET=&RID=UMNGA26401N&SHOW_LINKOUT=yes&SHOW_OVERVIEW=yes&STEP_NUMBER=&USE_ALIGNDB=true&ADV_VIEW=on&DISPLAY_SORT=1&HSP_SORT=1) | [**Total Score**](https://blast.ncbi.nlm.nih.gov/Blast.cgi?CMD=Get&ADV_VIEW=yes&ADV_VIEW=on&ALIGNDB_BATCH_ID=31534727&ALIGNDB_CGI_HOST=blast.st-va.ncbi.nlm.nih.gov&ALIGNDB_CGI_PATH=/ALIGNDB/alndb_asn.cgi&ALIGNDB_MASTER_ALIAS=SD_ALIGNDB_MASTER&ALIGNDB_MAX_ROWS=100&ALIGNDB_ORDER_CLAUSE=seq_evalue%20asc,aln_id%20asc&ALIGNDB_WHERE_CLAUSE=seq_evalue%20is%20not%20null&ALIGNMENTS=100&ALIGNMENT_VIEW=Pairwise&CONFIG_DESCR=ClustMemNbr,ClustComn,Ds,Sc,Ms,Ts,Cov,Eval,Idnt,AccLen,Acc&DATABASE_SORT=0&DESCRIPTIONS=100&DYNAMIC_FORMAT=on&FIRST_QUERY_NUM=0&FORMAT_NUM_ORG=1&FORMAT_OBJECT=Alignment&FORMAT_PAGE_TARGET=&FORMAT_TYPE=HTML&GET_SEQUENCE=yes&I_THRESH=&LINE_LENGTH=60&MASK_CHAR=2&MASK_COLOR=1&NUM_OVERVIEW=100&PAGE=MegaBlast&QUERY_INDEX=0&QUERY_NUMBER=0&RESULTS_PAGE_TARGET=&RID=UMNGA26401N&SHOW_LINKOUT=yes&SHOW_OVERVIEW=yes&STEP_NUMBER=&USE_ALIGNDB=true&ADV_VIEW=on&DISPLAY_SORT=2&HSP_SORT=1) | [**Query Cover**](https://blast.ncbi.nlm.nih.gov/Blast.cgi?CMD=Get&ADV_VIEW=yes&ADV_VIEW=on&ALIGNDB_BATCH_ID=31534727&ALIGNDB_CGI_HOST=blast.st-va.ncbi.nlm.nih.gov&ALIGNDB_CGI_PATH=/ALIGNDB/alndb_asn.cgi&ALIGNDB_MASTER_ALIAS=SD_ALIGNDB_MASTER&ALIGNDB_MAX_ROWS=100&ALIGNDB_ORDER_CLAUSE=seq_evalue%20asc,aln_id%20asc&ALIGNDB_WHERE_CLAUSE=seq_evalue%20is%20not%20null&ALIGNMENTS=100&ALIGNMENT_VIEW=Pairwise&CONFIG_DESCR=ClustMemNbr,ClustComn,Ds,Sc,Ms,Ts,Cov,Eval,Idnt,AccLen,Acc&DATABASE_SORT=0&DESCRIPTIONS=100&DYNAMIC_FORMAT=on&FIRST_QUERY_NUM=0&FORMAT_NUM_ORG=1&FORMAT_OBJECT=Alignment&FORMAT_PAGE_TARGET=&FORMAT_TYPE=HTML&GET_SEQUENCE=yes&I_THRESH=&LINE_LENGTH=60&MASK_CHAR=2&MASK_COLOR=1&NUM_OVERVIEW=100&PAGE=MegaBlast&QUERY_INDEX=0&QUERY_NUMBER=0&RESULTS_PAGE_TARGET=&RID=UMNGA26401N&SHOW_LINKOUT=yes&SHOW_OVERVIEW=yes&STEP_NUMBER=&USE_ALIGNDB=true&ADV_VIEW=on&DISPLAY_SORT=4&HSP_SORT=0) | [**Per. Ident**](https://blast.ncbi.nlm.nih.gov/Blast.cgi?CMD=Get&ADV_VIEW=yes&ADV_VIEW=on&ALIGNDB_BATCH_ID=31534727&ALIGNDB_CGI_HOST=blast.st-va.ncbi.nlm.nih.gov&ALIGNDB_CGI_PATH=/ALIGNDB/alndb_asn.cgi&ALIGNDB_MASTER_ALIAS=SD_ALIGNDB_MASTER&ALIGNDB_MAX_ROWS=100&ALIGNDB_ORDER_CLAUSE=seq_evalue%20asc,aln_id%20asc&ALIGNDB_WHERE_CLAUSE=seq_evalue%20is%20not%20null&ALIGNMENTS=100&ALIGNMENT_VIEW=Pairwise&CONFIG_DESCR=ClustMemNbr,ClustComn,Ds,Sc,Ms,Ts,Cov,Eval,Idnt,AccLen,Acc&DATABASE_SORT=0&DESCRIPTIONS=100&DYNAMIC_FORMAT=on&FIRST_QUERY_NUM=0&FORMAT_NUM_ORG=1&FORMAT_OBJECT=Alignment&FORMAT_PAGE_TARGET=&FORMAT_TYPE=HTML&GET_SEQUENCE=yes&I_THRESH=&LINE_LENGTH=60&MASK_CHAR=2&MASK_COLOR=1&NUM_OVERVIEW=100&PAGE=MegaBlast&QUERY_INDEX=0&QUERY_NUMBER=0&RESULTS_PAGE_TARGET=&RID=UMNGA26401N&SHOW_LINKOUT=yes&SHOW_OVERVIEW=yes&STEP_NUMBER=&USE_ALIGNDB=true&ADV_VIEW=on&DISPLAY_SORT=3&HSP_SORT=3) | [**Acc. Len**](https://blast.ncbi.nlm.nih.gov/Blast.cgi) |
| --- | --- | --- | --- | --- | --- | --- | --- |
| *bla*_KPC_ | SCL12139 | [MT949189.1](https://www.ncbi.nlm.nih.gov/nucleotide/MT949189.1?report=genbank&log$=nucltop&blast_rank=1&RID=UMDE8KN3013) | 56207 | 98012 | 85% | 100.00% | 42936 |
|  |  | [CP093467.1](https://www.ncbi.nlm.nih.gov/nucleotide/CP093467.1?report=genbank&log$=nucltop&blast_rank=2&RID=UMDE8KN3013) | 54878 | 1,71E+08 | 91% | 99.99% | 58125 |
|  |  | [CP093499.1](https://www.ncbi.nlm.nih.gov/nucleotide/CP093499.1?report=genbank&log$=nucltop&blast_rank=3&RID=UMDE8KN3013) | 54872 | 1,69E+08 | 91% | 99.99% | 58118 |
|  |  | [CP135187.1](https://www.ncbi.nlm.nih.gov/nucleotide/CP135187.1?report=genbank&log$=nucltop&blast_rank=4&RID=UMDE8KN3013) | 52392 | 1,72E+08 | 91% | 99.99% | 54404 |
|  |  | [CP019026.1](https://www.ncbi.nlm.nih.gov/nucleotide/CP019026.1?report=genbank&log$=nucltop&blast_rank=5&RID=UMDE8KN3013) | 39403 | 1,77E+08 | 91% | 99.99% | 59373 |
|  | SCL13680 | [CP093499.1](https://www.ncbi.nlm.nih.gov/nucleotide/CP093499.1?report=genbank&log$=nucltop&blast_rank=1&RID=UMFBH34F013) | 51637 | 1,95E+08 | 98% | 99.98% | 58118 |
|  |  | [CP093467.1](https://www.ncbi.nlm.nih.gov/nucleotide/CP093467.1?report=genbank&log$=nucltop&blast_rank=2&RID=UMFBH34F013) | 51631 | 1,97E+08 | 98% | 99.97% | 58125 |
|  |  | [CP052348.1](https://www.ncbi.nlm.nih.gov/nucleotide/CP052348.1?report=genbank&log$=nucltop&blast_rank=3&RID=UMFBH34F013) | 51158 | 1,85E+08 | 87% | 99.98% | 55469 |
|  |  | [CP044159.1](https://www.ncbi.nlm.nih.gov/nucleotide/CP044159.1?report=genbank&log$=nucltop&blast_rank=4&RID=UMFBH34F013) | 51158 | 1,86E+08 | 87% | 99.98% | 59694 |
|  |  | [CP135501.1](https://www.ncbi.nlm.nih.gov/nucleotide/CP135501.1?report=genbank&log$=nucltop&blast_rank=5&RID=UMFBH34F013) | 51158 | 1,86E+08 | 87% | 99.98% | 55403 |
|  | SCL13938 | [CP093499.1](https://www.ncbi.nlm.nih.gov/nucleotide/CP093499.1?report=genbank&log$=nucltop&blast_rank=1&RID=UMFN9ESY016) | 1,04E+08 | 1,38E+08 | 100% | 99.97% | 58118 |
|  |  | [CP093467.1](https://www.ncbi.nlm.nih.gov/nucleotide/CP093467.1?report=genbank&log$=nucltop&blast_rank=2&RID=UMFN9ESY016) | 1,04E+08 | 1,39E+08 | 100% | 99.97% | 58125 |
|  |  | [CP135187.1](https://www.ncbi.nlm.nih.gov/nucleotide/CP135187.1?report=genbank&log$=nucltop&blast_rank=3&RID=UMFN9ESY016) | 80786 | 1,20E+08 | 88% | 99.99% | 54404 |
|  |  | [MT949189.1](https://www.ncbi.nlm.nih.gov/nucleotide/MT949189.1?report=genbank&log$=nucltop&blast_rank=4&RID=UMFN9ESY016) | 78889 | 90841 | 76% | 100.00% | 42936 |
|  |  | [CP019026.1](https://www.ncbi.nlm.nih.gov/nucleotide/CP019026.1?report=genbank&log$=nucltop&blast_rank=5&RID=UMFN9ESY016) | 59319 | 1,44E+08 | 100% | 99.99% | 59373 |
|  | SCL13636 | [CP093482.1](https://www.ncbi.nlm.nih.gov/nucleotide/CP093482.1?report=genbank&log$=nucltop&blast_rank=1&RID=UME8JV8P01N) | 1,21E+08 | 3,46E+08 | 85% | 99.98% | 178685 |
|  |  | [CP061833.1](https://www.ncbi.nlm.nih.gov/nucleotide/CP061833.1?report=genbank&log$=nucltop&blast_rank=2&RID=UME8JV8P01N) | 1,21E+08 | 3,65E+08 | 87% | 99.98% | 193300 |
|  |  | [CP139936.1](https://www.ncbi.nlm.nih.gov/nucleotide/CP139936.1?report=genbank&log$=nucltop&blast_rank=3&RID=UME8JV8P01N) | 1,21E+08 | 3,39E+08 | 85% | 99.98% | 167501 |
|  |  | [CP098142.1](https://www.ncbi.nlm.nih.gov/nucleotide/CP098142.1?report=genbank&log$=nucltop&blast_rank=4&RID=UME8JV8P01N) | 1,21E+08 | 3,50E+08 | 86% | 99.98% | 194865 |
|  |  | [CP098137.1](https://www.ncbi.nlm.nih.gov/nucleotide/CP098137.1?report=genbank&log$=nucltop&blast_rank=5&RID=UME8JV8P01N) | 1,21E+08 | 3,50E+08 | 86% | 99.98% | 194864 |
|  | SCL14225 | [CP056338.1](https://www.ncbi.nlm.nih.gov/nucleotide/CP056338.1?report=genbank&log$=nucltop&blast_rank=1&RID=UMFZJ4X1016) | 32282 | 1,56E+08 | 85% | 99.23% | 98779 |
|  |  | [CP135187.1](https://www.ncbi.nlm.nih.gov/nucleotide/CP135187.1?report=genbank&log$=nucltop&blast_rank=2&RID=UMFZJ4X1016) | 12286 | 12286 | 6% | 99.98% | 54404 |
|  |  | [CP093499.1](https://www.ncbi.nlm.nih.gov/nucleotide/CP093499.1?report=genbank&log$=nucltop&blast_rank=3&RID=UMFZJ4X1016) | 12286 | 12286 | 6% | 99.98% | 58118 |
|  |  | [CP093487.1](https://www.ncbi.nlm.nih.gov/nucleotide/CP093487.1?report=genbank&log$=nucltop&blast_rank=4&RID=UMFZJ4X1016) | 12286 | 12286 | 6% | 99.98% | 90276 |
|  |  | [CP093467.1](https://www.ncbi.nlm.nih.gov/nucleotide/CP093467.1?report=genbank&log$=nucltop&blast_rank=5&RID=UMFZJ4X1016) | 12286 | 12286 | 6% | 99.98% | 58125 |
|  | SCL16432 | [CP044159.1](https://www.ncbi.nlm.nih.gov/nucleotide/CP044159.1?report=genbank&log$=nucltop&blast_rank=1&RID=UMGCEN40016) | 51157 | 1,86E+08 | 85% | 100.00% | 59694 |
|  |  | [CP135501.1](https://www.ncbi.nlm.nih.gov/nucleotide/CP135501.1?report=genbank&log$=nucltop&blast_rank=2&RID=UMGCEN40016) | 51157 | 1,86E+08 | 85% | 100.00% | 55403 |
|  |  | [CP135452.1](https://www.ncbi.nlm.nih.gov/nucleotide/CP135452.1?report=genbank&log$=nucltop&blast_rank=3&RID=UMGCEN40016) | 51157 | 1,94E+08 | 90% | 100.00% | 85586 |
|  |  | [LC663730.1](https://www.ncbi.nlm.nih.gov/nucleotide/LC663730.1?report=genbank&log$=nucltop&blast_rank=4&RID=UMGCEN40016) | 51157 | 1,84E+08 | 85% | 100.00% | 55403 |
|  |  | [LC663726.1](https://www.ncbi.nlm.nih.gov/nucleotide/LC663726.1?report=genbank&log$=nucltop&blast_rank=5&RID=UMGCEN40016) | 51157 | 1,84E+08 | 85% | 100.00% | 55403 |
|  |  | [CP049024.1](https://www.ncbi.nlm.nih.gov/nucleotide/CP049024.1?report=genbank&log$=nucltop&blast_rank=6&RID=UMGCEN40016) | 51157 | 1,86E+08 | 85% | 100.00% | 55421 |
|  |  | [CP048699.1](https://www.ncbi.nlm.nih.gov/nucleotide/CP048699.1?report=genbank&log$=nucltop&blast_rank=7&RID=UMGCEN40016) | 51157 | 1,86E+08 | 85% | 100.00% | 55421 |
|  |  | [CP093467.1](https://www.ncbi.nlm.nih.gov/nucleotide/CP093467.1?report=genbank&log$=nucltop&blast_rank=8&RID=UMGCEN40016) | 51092 | 1,94E+08 | 93% | 99.96% | 58125 |
|  |  | [CP093499.1](https://www.ncbi.nlm.nih.gov/nucleotide/CP093499.1?report=genbank&log$=nucltop&blast_rank=9&RID=UMGCEN40016) | 51046 | 1,92E+08 | 93% | 99.94% | 58118 |
|  | SCL16743 | [CP136407.1](https://www.ncbi.nlm.nih.gov/nucleotide/CP136407.1?report=genbank&log$=nucltop&blast_rank=1&RID=UMHMDJBY013) | 1,25E+08 | 1,97E+08 | 100% | 100.00% | 113639 |
|  |  | [CP067847.1](https://www.ncbi.nlm.nih.gov/nucleotide/CP067847.1?report=genbank&log$=nucltop&blast_rank=2&RID=UMHMDJBY013) | 1,25E+08 | 2,03E+08 | 90% | 100.00% | 137297 |
|  |  | [CP011991.1](https://www.ncbi.nlm.nih.gov/nucleotide/CP011991.1?report=genbank&log$=nucltop&blast_rank=3&RID=UMHMDJBY013) | 1,25E+08 | 1,97E+08 | 100% | 99.99% | 113638 |
|  |  | [CP054266.1](https://www.ncbi.nlm.nih.gov/nucleotide/CP054266.1?report=genbank&log$=nucltop&blast_rank=4&RID=UMHMDJBY013) | 1,25E+08 | 1,77E+08 | 90% | 99.96% | 89626 |
|  |  | [MW650887.1](https://www.ncbi.nlm.nih.gov/nucleotide/MW650887.1?report=genbank&log$=nucltop&blast_rank=5&RID=UMHMDJBY013) | 1,25E+08 | 2,30E+08 | 100% | 99.96% | 238655 |
|  |  | [KJ146687.1](https://www.ncbi.nlm.nih.gov/nucleotide/KJ146687.1?report=genbank&log$=nucltop&blast_rank=11&RID=UMHMDJBY013) | 1,24E+08 | 1,97E+08 | 100% | 100.00% | 113639 |
|  |  | [KJ146689.1](https://www.ncbi.nlm.nih.gov/nucleotide/KJ146689.1?report=genbank&log$=nucltop&blast_rank=12&RID=UMHMDJBY013) | 1,24E+08 | 1,99E+08 | 100% | 100.00% | 114464 |
|  |  | [CP011986.1](https://www.ncbi.nlm.nih.gov/nucleotide/CP011986.1?report=genbank&log$=nucltop&blast_rank=13&RID=UMHMDJBY013) | 1,24E+08 | 1,96E+08 | 100% | 99.99% | 113639 |
|  |  | [KJ146688.1](https://www.ncbi.nlm.nih.gov/nucleotide/KJ146688.1?report=genbank&log$=nucltop&blast_rank=14&RID=UMHMDJBY013) | 1,24E+08 | 1,97E+08 | 100% | 99.99% | 99142 |
| *bla*_NDM_ | NDM7_SCL12139 | [KX023261.1](https://www.ncbi.nlm.nih.gov/nucleotide/KX023261.1?report=genbank&log$=nucltop&blast_rank=3&RID=UMJ44EZ6016) | 56695 | 85577 | 100% | 99.99% | 46254 |
|  |  | [CP141747.1](https://www.ncbi.nlm.nih.gov/nucleotide/CP141747.1?report=genbank&log$=nucltop&blast_rank=4&RID=UMJ44EZ6016) | 56695 | 83326 | 97% | 99.99% | 45122 |
|  |  | [KX214669.1](https://www.ncbi.nlm.nih.gov/nucleotide/KX214669.1?report=genbank&log$=nucltop&blast_rank=5&RID=UMJ44EZ6016) | 56695 | 85227 | 100% | 99.99% | 46161 |
|  |  | [CP093492.1](https://www.ncbi.nlm.nih.gov/nucleotide/CP093492.1?report=genbank&log$=nucltop&blast_rank=6&RID=UMJ44EZ6016) | 56695 | 94931 | 100% | 99.99% | 68131 |
|  |  | [KM400601.1](https://www.ncbi.nlm.nih.gov/nucleotide/KM400601.1?report=genbank&log$=nucltop&blast_rank=7&RID=UMJ44EZ6016) | 56695 | 85594 | 100% | 99.99% | 46253 |
|  |  | [KP776609.1](https://www.ncbi.nlm.nih.gov/nucleotide/KP776609.1?report=genbank&log$=nucltop&blast_rank=8&RID=UMJ44EZ6016) | 56695 | 83311 | 97% | 99.99% | 45122 |
|  |  | [OW967968.1](https://www.ncbi.nlm.nih.gov/nucleotide/OW967968.1?report=genbank&log$=nucltop&blast_rank=9&RID=UMJ44EZ6016) | 56695 | 85242 | 100% | 99.99% | 46161 |
|  | NDM7_SCL13636 | [LC536680.1](https://www.ncbi.nlm.nih.gov/nucleotide/LC536680.1?report=genbank&log$=nucltop&blast_rank=1&RID=UMKM8GTS016) | 42145 | 88485 | 81% | 99.98% | 46161 |
|  |  | [MN061454.1](https://www.ncbi.nlm.nih.gov/nucleotide/MN061454.1?report=genbank&log$=nucltop&blast_rank=2&RID=UMKM8GTS016) | 42145 | 88145 | 80% | 99.98% | 53023 |
|  |  | [CP050161.1](https://www.ncbi.nlm.nih.gov/nucleotide/CP050161.1?report=genbank&log$=nucltop&blast_rank=4&RID=UMKM8GTS016) | 42145 | 90002 | 81% | 99.98% | 54035 |
|  |  | [CP028786.2](https://www.ncbi.nlm.nih.gov/nucleotide/CP028786.2?report=genbank&log$=nucltop&blast_rank=7&RID=UMKM8GTS016) | 42145 | 90032 | 81% | 99.98% | 54035 |
|  |  | [CP031884.1](https://www.ncbi.nlm.nih.gov/nucleotide/CP031884.1?report=genbank&log$=nucltop&blast_rank=8&RID=UMKM8GTS016) | 42145 | 87400 | 80% | 99.98% | 105259 |
|  |  | [MH917283.1](https://www.ncbi.nlm.nih.gov/nucleotide/MH917283.1?report=genbank&log$=nucltop&blast_rank=9&RID=UMKM8GTS016) | 42145 | 90018 | 81% | 99.98% | 54035 |
|  |  | [MH105050.1](https://www.ncbi.nlm.nih.gov/nucleotide/MH105050.1?report=genbank&log$=nucltop&blast_rank=11&RID=UMKM8GTS016) | 42145 | 95550 | 81% | 99.98% | 216895 |
|  | NDM7_SCL13674 | [CP084538.1](https://www.ncbi.nlm.nih.gov/nucleotide/CP084538.1?report=genbank&log$=nucltop&blast_rank=1&RID=UMMD0MGF013) | 85183 | 1,72E+08 | 100% | 99.98% | 92324 |
|  |  | [KU167608.1](https://www.ncbi.nlm.nih.gov/nucleotide/KU167608.1?report=genbank&log$=nucltop&blast_rank=2&RID=UMMD0MGF013) | 85028 | 86024 | 100% | 99.98% | 46161 |
|  |  | [KU167609.1](https://www.ncbi.nlm.nih.gov/nucleotide/KU167609.1?report=genbank&log$=nucltop&blast_rank=3&RID=UMMD0MGF013) | 85017 | 86012 | 100% | 99.97% | 46161 |
|  |  | [AP019679.1](https://www.ncbi.nlm.nih.gov/nucleotide/AP019679.1?report=genbank&log$=nucltop&blast_rank=4&RID=UMMD0MGF013) | 85015 | 86011 | 100% | 99.97% | 46158 |
|  |  | [KF220657.1](https://www.ncbi.nlm.nih.gov/nucleotide/KF220657.1?report=genbank&log$=nucltop&blast_rank=5&RID=UMMD0MGF013) | 85011 | 86328 | 100% | 99.97% | 46253 |
|  |  | [KT824791.1](https://www.ncbi.nlm.nih.gov/nucleotide/KT824791.1?report=genbank&log$=nucltop&blast_rank=6&RID=UMMD0MGF013) | 85009 | 86005 | 100% | 99.97% | 46164 |
|  | NDM7_SCL13938 | [CP084538.1](https://www.ncbi.nlm.nih.gov/nucleotide/CP084538.1?report=genbank&log$=nucltop&blast_rank=1&RID=UMMRWY8B016) | 85205 | 1,72E+08 | 100% | 99.98% | 92324 |
|  |  | [KU167608.1](https://www.ncbi.nlm.nih.gov/nucleotide/KU167608.1?report=genbank&log$=nucltop&blast_rank=2&RID=UMMRWY8B016) | 85050 | 86046 | 100% | 99.98% | 46161 |
|  |  | [KU167609.1](https://www.ncbi.nlm.nih.gov/nucleotide/KU167609.1?report=genbank&log$=nucltop&blast_rank=3&RID=UMMRWY8B016) | 85039 | 86035 | 100% | 99.98% | 46161 |
|  |  | [AP019679.1](https://www.ncbi.nlm.nih.gov/nucleotide/AP019679.1?report=genbank&log$=nucltop&blast_rank=4&RID=UMMRWY8B016) | 85037 | 86033 | 100% | 99.98% | 46158 |
|  |  | [KF220657.1](https://www.ncbi.nlm.nih.gov/nucleotide/KF220657.1?report=genbank&log$=nucltop&blast_rank=5&RID=UMMRWY8B016) | 85033 | 86350 | 100% | 99.98% | 46253 |
|  |  | [KT824791.1](https://www.ncbi.nlm.nih.gov/nucleotide/KT824791.1?report=genbank&log$=nucltop&blast_rank=6&RID=UMMRWY8B016) | 85031 | 86027 | 100% | 99.98% | 46164 |
|  | NDM7_SCL14225 | [LR697126.1](https://www.ncbi.nlm.nih.gov/nucleotide/LR697126.1?report=genbank&log$=nucltop&blast_rank=1&RID=UMMWVV88016) | 56497 | 88380 | 97% | 100.00% | 44885 |
|  |  | [CP023260.1](https://www.ncbi.nlm.nih.gov/nucleotide/CP023260.1?report=genbank&log$=nucltop&blast_rank=2&RID=UMMWVV88016) | 56497 | 87365 | 95% | 100.00% | 44106 |
|  |  | [KX023261.1](https://www.ncbi.nlm.nih.gov/nucleotide/KX023261.1?report=genbank&log$=nucltop&blast_rank=3&RID=UMMWVV88016) | 56497 | 91674 | 100% | 100.00% | 46254 |
|  |  | [CP141747.1](https://www.ncbi.nlm.nih.gov/nucleotide/CP141747.1?report=genbank&log$=nucltop&blast_rank=4&RID=UMMWVV88016) | 56497 | 89426 | 97% | 100.00% | 45122 |
|  |  | [KX214669.1](https://www.ncbi.nlm.nih.gov/nucleotide/KX214669.1?report=genbank&log$=nucltop&blast_rank=5&RID=UMMWVV88016) | 56497 | 91314 | 100% | 100.00% | 46161 |
|  |  | [CP093492.1](https://www.ncbi.nlm.nih.gov/nucleotide/CP093492.1?report=genbank&log$=nucltop&blast_rank=6&RID=UMMWVV88016) | 56497 | 1,05E+08 | 100% | 100.00% | 68131 |
|  |  | [KM400601.1](https://www.ncbi.nlm.nih.gov/nucleotide/KM400601.1?report=genbank&log$=nucltop&blast_rank=7&RID=UMMWVV88016) | 56497 | 91694 | 100% | 100.00% | 46253 |
|  | NDM7_SCL16432 | [KX023261.1](https://www.ncbi.nlm.nih.gov/nucleotide/KX023261.1?report=genbank&log$=nucltop&blast_rank=3&RID=UMN3UDD1016) | 56708 | 85590 | 100% | 100.00% | 46254 |
|  |  | [KX214669.1](https://www.ncbi.nlm.nih.gov/nucleotide/KX214669.1?report=genbank&log$=nucltop&blast_rank=5&RID=UMN3UDD1016) | 56708 | 85240 | 100% | 100.00% | 46161 |
|  |  | [CP093492.1](https://www.ncbi.nlm.nih.gov/nucleotide/CP093492.1?report=genbank&log$=nucltop&blast_rank=6&RID=UMN3UDD1016) | 56708 | 94944 | 100% | 100.00% | 68131 |
|  |  | [KM400601.1](https://www.ncbi.nlm.nih.gov/nucleotide/KM400601.1?report=genbank&log$=nucltop&blast_rank=7&RID=UMN3UDD1016) | 56708 | 85607 | 100% | 100.00% | 46253 |
|  | NDM7_SCL16743 | [CP106917.1](https://www.ncbi.nlm.nih.gov/nucleotide/CP106917.1?report=genbank&log$=nucltop&blast_rank=1&RID=UMNGA26401N) | 15535 | 44053 | 82% | 99.95% | 47483 |
|  |  | [MH523639.1](https://www.ncbi.nlm.nih.gov/nucleotide/MH523639.1?report=genbank&log$=nucltop&blast_rank=2&RID=UMNGA26401N) | 11769 | 39218 | 82% | 99.98% | 46161 |
|  |  | [CP035125.1](https://www.ncbi.nlm.nih.gov/nucleotide/CP035125.1?report=genbank&log$=nucltop&blast_rank=3&RID=UMNGA26401N) | 11769 | 39217 | 82% | 99.98% | 46161 |
|  |  | [CP025215.1](https://www.ncbi.nlm.nih.gov/nucleotide/CP025215.1?report=genbank&log$=nucltop&blast_rank=4&RID=UMNGA26401N) | 11769 | 55741 | 82% | 99.98% | 68637 |
|  |  | [KX214669.1](https://www.ncbi.nlm.nih.gov/nucleotide/KX214669.1?report=genbank&log$=nucltop&blast_rank=5&RID=UMNGA26401N) | 11769 | 39217 | 82% | 99.98% | 46161 |
|  |  | [CP093504.1](https://www.ncbi.nlm.nih.gov/nucleotide/CP093504.1?report=genbank&log$=nucltop&blast_rank=6&RID=UMNGA26401N) | 11769 | 39218 | 82% | 99.98% | 46161 |

Blastn was performed using the tool https://blast.ncbi.nlm.nih.gov/Blast.cgi. The most representative results for each plasmid identified in this research are shown. Accession: a unique identifier assigned to records in the NCBI databases. Max Score: the highest alignment score calculated from the sum of the rewards for

matched nucleotides and penalities for mismatches and gaps. Total Score: the sum of alignment scores of all segments from the same subject sequence. Query Cover: the percent of the query length that is included in the aligned segments. Ident: the highest percent identity for a set of aligned segments to the same subject sequence. Acc. Len: the number of nucleotides or amino acids in the result sequence identified by the accession number.

**Supplementary Table 3**. Number of SNPs between the first isolated and consecutive *E. coli* isolates recovered from patients 3 and 4 respectively.

| ID Patient | Nº. SNP *vs*. First isolate | | |
| --- | --- | --- | --- |
|  | Isolate 2 | Isolate 3 | Isolate 4 |
| Patient 3 | 63 | - | - |
| Patient 4 | 96 | 97 | 94 |


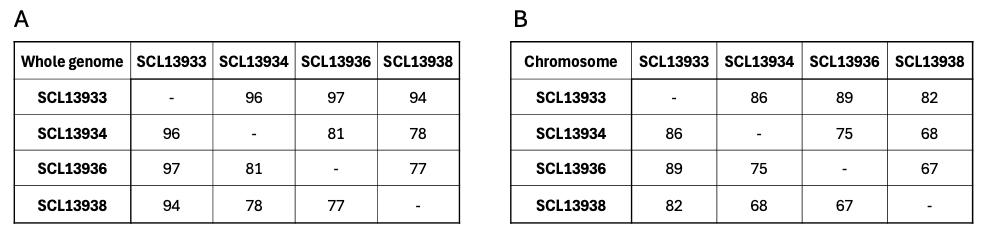


**Supplementary Figure 1**. Number of SNPs among four consecutive *E. coli* isolates recovered from patient 4. A. SNPs found when the complete genomes were compared, including chromosome and plasmids. B. SNPs found in the biggest contig obtained by long-read sequencing.

**Supplementary Figure 2**. Schematic representation of plasmids. **A.** *bla*_NDM-7_ gene, and **B.** *bla*_KPC-2_ gene. Dotted colored lines correspond to those in Figure 3A. Blue arrows represent transposable elements (TE), red arrows represent antibiotic resistance genes, green arrow represent *bla*_KPC-2_ and *bla*_NDM-7_ gene, and orange represent *bla*_KPC-3_.
